# Egg genotyping reveals the possibility of patent *Ancylostoma caninum* infection in human intestine

**DOI:** 10.1038/s41598-020-59874-8

**Published:** 2020-02-20

**Authors:** Luis Fernando Viana Furtado, Lucas Teixeira de Oliveira Dias, Thais de Oliveira Rodrigues, Vivian Jordania da Silva, Valéria Nayara Gomes Mendes de Oliveira, Élida Mara Leite Rabelo

**Affiliations:** 1 Universidade do Estado de Minas Gerais, Avenida Juca Stockler, 1130, CEP 37900-106, Nossa Sra. das Gracas, Passos, Minas Gerais Brazil; 2 Universidade Federal de Minas Gerais, Instituto de Ciências Biológicas, Avenida Presidente Antônio Carlos, 6627, Departamento de Parasitologia, L4 237, Laboratório de Parasitologia Molecular, Pampulha, CEP 31270-901, Belo Horizonte, Minas Gerais Brazil

**Keywords:** Environmental biotechnology, Evolutionary developmental biology

## Abstract

Hookworms are intestinal parasites that cause major public health problems, especially in developing countries. To differentiate eggs from different hookworm species, it is necessary to use molecular methodologies, since the eggs are morphologically similar. Here, we performed the molecular identification of single hookworm eggs from six Brazilian states. Of the 634 eggs individually analyzed, 98.1% (622/634) represented *Necator americanus*, and surprisingly, 1.9% (12/634 eggs from the same patient) represented *Ancylostoma caninum*. DNA analysis of the *A. caninum*-positive stool sample revealed no contamination with animal feces. This is the first report of the presence of *A. caninum* eggs in human feces, which may have a direct implication for the epidemiology of hookworm infection caused by this species. This suggests the need for special attention regarding prophylaxis, as different reservoirs, previously not described, may have great relevance for the spread of *A. caninum*.

## Introduction

Hookworms affect almost 500 million people worldwide, mainly in developing countries, resulting in a global disease burden of 3.5 million disability-adjusted life years^1,2^. Human ancylostomiasis is caused mainly by the species *Ancylostoma duodenale* and *Necator americanus*; *A. braziliense* parasitize both canids and felids while *A. tubaeforme* and *A. caninum* parasitize mainly felids and canids, respectively^3,4^. Among the zoonotic hookworm species, until now, only *A. ceylanicum* have been shown to cause patent human infections, for which dogs and cats can be reservoirs. These parasites indirectly may cause anaemia, diarrhea, intestinal cramps, and the delayed cognitive and physical development of the host^5,6^.

Classically, *A. braziliense* and *A. caninum* larvae have been characterized as etiological agents of skin infections in humans known as *larva migrans*^4^. *Ancylostoma caninum* also has the ability to cause eosinophilic enteritis in humans^7^, and there have been a few reports of the natural parasitism of adult worms of this species in human intestines^8,9^. However, none of these studies have reported sexually mature worms, so egg production has never been detected. In fact, experimental infections in humans with *A. caninum* resulted in intermittent abdominal pain and eosinophilia, but eggs were not observed in feces at any time^10^. Nevertheless, George and coworkers^11^ detected DNA from *A. caninum* in human feces, suggesting that the role of animals as reservoirs of hookworms in humans may be underestimated and should be investigated.

Since hookworms have morphologically similar eggs, many studies have used standardized molecular techniques for hookworm species differentiation in dogs^12,13^, cats^14^ and humans^15,16^. Additionally, because the eggs are identical, the true prevalence of the hookworm species that affect humans is undetermined in most regions in the world in areas where these parasites are endemic. Therefore, in this work, we aimed to molecularly identify the species of hookworms parasitizing humans. This is of crucial importance because different populations of parasite species are a factor that may affect the prevention strategies used against parasites, since each species may have its own hosts, routes of transmission and pathogenesis^17,18^. In addition, such screenings may help to answer controversial questions about zoonoses^11^. Here, we performed the molecular identification of individual hookworm eggs from six Brazilian states. For the first time, we report the presence of *A. caninum* eggs in human fecal samples, which indicates an important and neglected epidemiological issue.

## Results

**Figure 1:**
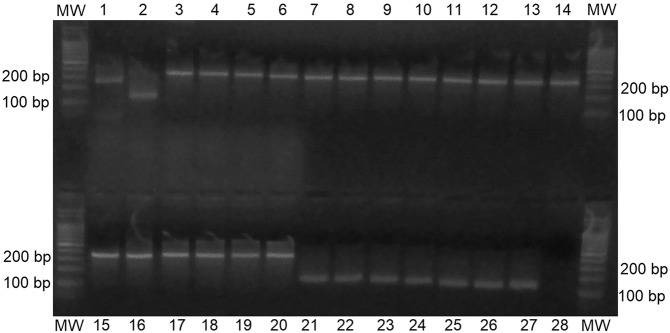
Representative PCR results from the molecular identification of single hookworm eggs. In lanes 1 and 2, controls were used (lane 1: *N. americanus* - 250 bp; lane 2: *Ancylostoma* spp. - 130 bp). Lanes 3 to 27 show the PCR products from single egg DNA (3 to 20: *N. americanus*; 21 to 27: *Ancylostoma* spp.). Lane 28 corresponds to the blank control for the reactions. The image shows an agarose gel (1%) that was stained with GelRed™ (Biotium, USA). MW: 100 bp molecular weight.

**Figure 2:**
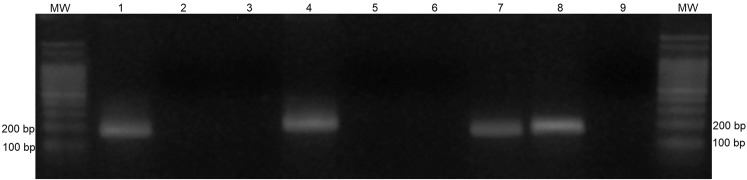
PCR results from the molecular identification of the host and the assessment of possible contamination. In lanes 1 to 3, 4 to 6, and 7 to 9, primers were used for amplification of cat (*F. catus* – 167 bp), dog (*C. familiaris* – 193 bp) and human (*H. sapiens* – 162 bp) DNA, respectively. In lanes 1, 4 and 7, positive controls were used (lane 1: *F. catus*; lane 4: *C. familiaris*; lane 7: *H. sapiens*). Lanes 2, 5 and 8 show the PCR products from DNA extracted from feces positive for *A. caninum*. Lanes 3, 6 and 9 correspond to the blank controls for the reactions. The image shows an agarose gel (1%) that was stained with GelRed™ (Biotium, USA). MW: 100 bp molecular weight. The lack of fecal DNA amplification in lanes 2 and 5 indicates there was no contamination with animal fecal samples.

We analyzed 634 single hookworm eggs from fecal samples from 53 humans from six Brazilian states by conventional PCR. Of all the eggs analyzed, 98.1% (622/634) were identified as *N. americanus*, and 1.9% (12/634) were identified as *Ancylostoma* spp. (all of which were obtained from the same single-parasitized individual from Minas Gerais). The sequencing of the samples identified as *Ancylostoma* spp. revealed that these eggs were from *A. caninum*, demonstrating 100% of similarity to the *A. caninum* sequences available in the GenBank database (Accession numbers: KP844730.1; DQ438075.1; DQ438071.1). To determine the presence of possible contamination from dog and cat samples, DNA extraction of the *A. caninum*-positive feces was performed. By using conventional PCR and subsequent sequencing, it was possible to determine that the sample had only human DNA; therefore, there was no evidence of contamination with animal feces. Figures 1 and 2 show representative agarose gels from the analyses of the molecular identification of hookworms and hosts, respectively.

## Discussion

Hookworms are important nematodes in both human and veterinary medicine^3,4^. Since eggs of different hookworm species have similar morphology, diagnosis is routinely and generically provided as hookworm only^5^. Knowledge of the species in question is fundamental for determining the true prevalence of various parasites as well as for adopting control measures, such as vaccine production and coverage^18,19^. Here, we molecularly identified hookworm species collected from naturally infected humans from six Brazilian states.

Many studies have performed the molecular identification of hookworms from stool samples of dogs^13^, cats^14^ and humans^15,16^. These studies have performed analyses using different molecular techniques, such as restriction fragment length polymorphism (RFLP-PCR)^12,20^, high resolution melting (HRM)^21^, quantitative real-time multiplex PCR^22^, single-strand conformation polymorphism (SSCP)^23^ and sequencing^24,25^. All of these studies performed analyses of adult worms and egg/larvae pools. We performed our analyses by conventional PCR by using a method previously standardized by Sahimin and coworkers^16^, with some modifications. To the best of our knowledge, this is the first study to identify hookworm species from single eggs. This is important because individual analysis allows the accurate detection of species, whereas, depending on the sensitivity of the technique, egg/larvae pool analysis can detect only the most prevalent species in the sample. In addition, obtaining adult worms for morphological analysis is unfeasible for obvious reasons.

Of the 634 eggs evaluated, 98.1% (622/634) were identified as *N. americanus*. In fact, according to Loukas and coworkers^5^, *N. americanus* is the predominant human hookworm, which globally accounts for the majority of patent human hookworm cases and is especially common in the Americas. These data corroborate the molecular analyses of Monteiro and coworkers^25^, who evaluated hookworm populations in humans in northeastern Brazil. Brooker and coworkers^26^, by using morphological analysis, observed that all adult hookworms recovered from populations in southeastern Brazil were *N. americanus*, while Marzochi and Chieffi^27^, by using the morphological analysis of larvae obtained after fecal culture, observed the presence of *A. duodenale* in 14.2% of the analyzed samples. Studies in other South American countries, such as Ecuador, have also described *A. duodenale* in the intestines of humans^28^. In the present study, no *A. duodenale* eggs were detected. Biological characteristics linked to the development of *N. americanus* explain why this species is the most prevalent. The eggs of this species do not develop at temperatures below 15 °C, and the larvae prefer shady, moist areas with temperatures at or above 30 °C^29^.

*Ancylostoma ceylanicum*, primarily described as a dog and cat nematode, is highly prevalent in humans in Southeast Asia, including Malaysia and Indonesia^30^. Although it has been consistently used as an experimental model for studies in Brazil^31,32^, *A. ceylanicum* has never been described in animal or human populations in this country. However, the migratory flow of tourists and workers to Brazil observed in recent decades must be carefully analyzed, since this migration has already been responsible for introducing *A. ceylanicum* species to countries in which it had not been previously described^33^.

Although many studies conducted worldwide have revealed a high prevalence of *A. caninum* in canids and felids^13,34^, the infection by this species of humans has so far been restricted to skin infections that have mainly been acquired in parks and coastal regions^35^. Nevertheless, our results reveal for the first time the presence of *A. caninum* eggs in human feces. This may have a direct implication for the epidemiology of human parasitism by this species, since the source of infection could not be restricted to infected animals only. This should be critically studied further, especially since control strategies have been focused on classically described hosts (dogs and cats) in terms of vaccine development and coverage. Indeed, in recent decades, the target glutathione-s-transferase-1 has been studied as a vaccine antigen for *N. americanus* (Na-GST-1)^36^ and *A. caninum* (Ac-GST-1)^37^. Although this vaccine has resulted in a decreased number of adult worms and fecal eggs in dogs^37^, tests of the efficacy of this vaccine in humans have never been performed^36^. Because vaccine targets are species-specific, variation in hookworm species in a geographical region may negatively influence vaccine efficacy. In fact, regional variability may even impact anthelmintic efficacy; mutations involved in drug resistance in *A. caninum* collected in the field from different regions of Brazil have been described by our group^38^.

Although *A. caninum* eggs have never been observed in human feces, George and coworkers^11^ detected DNA from *A. caninum* in a fecal sample. These studies did not allow us to conclude that the eggs present in the samples were from this species, since the analyses were made based on DNA extraction from feces that also had *N. americanus* (which has eggs similar to those of *A. caninum*). According to previous authors, immature forms in the intestines of humans could release DNA into the human intestine, which would be detected by molecular methods. Our analyses, having been performed using DNA from a single egg, allow us to state with confidence that 1.9% (12/634) of the eggs represented *Ancylostoma* spp., which were identified as *A. caninum* by sequencing.

One hypothesis that could be proposed to explain the presence of *A. caninum* eggs in human feces would be the ingestion of eggs present in contaminated water and food and the consequent intact excretion of these eggs in feces. This has already been demonstrated in dogs with coprophagic habits, which can excrete *Eimeria* spp. and non-dog typical helminth eggs^39,40^. This is especially common for plant nematodes that are commonly ingested during human feeding, such as a parasite of the genus *Meloidogyne*^41^, causing infected patients to present with eggs in their feces without completion of the parasite cycle. However, hookworm eggs have a single and very thin membrane that would most likely be destroyed during passage through the stomach of humans^42^. Thus, the morphological characteristics of *A. caninum* eggs make this hypothesis strongly untenable. In addition, by observing the stage of egg development under optical microscopy, there was an absence of embryonated eggs in the feces. In case of the eggs simply being in transit through the gastrointestinal tract, it would seem probable that they would already be embryonated (see Supplementary Fig. S1).

*A. caninum* eggs found in a single patient sample could suggest contamination with fecal material from dogs and cats. However, DNA extraction from stools followed by molecular analyses to identify possible contaminants, performed in duplicate, revealed that only human DNA was found in the sample. This suggests that the eggs found did not come from the feces of animals infected with *A. caninum*. It has also to be considered that the potential uptake of animals *A. caninum* eggs does not necessarily mean that relevant amounts of animals faeces need to be taken up, since eggs may have been attached to food. In fact, many studies report the presence of hookworm eggs in food^43,44^ and also in water^45^. However, this a very low probability since twelve *A. caninum* eggs were found in the respective patient. In addition, some wildlife may be considered *A. caninum* hosts. Our analyzes from the feces of the patient with *A. caninum* would not be able to detect DNA from some of these animals, such as coyotes^46^. However, contamination of samples with material from these animals seems unlikely.

Unfortunately, we do not have any information regarding the patients, that include data related to the lifestyle habits of individuals, such as domesticated animals and work activities. Since dogs and cats are considered the main definitive hosts of *A. caninum*, living with infected animals could be a risk factor for the acquisition of the parasite^20^, either by causing larva migrans infections or by its development in the human intestine. In addition, work activities such as farm workers and other services that require contact with the soil can be considered a risk factor^34^. It is also important to consider the possibility of human host immunosuppression, since the absence of an effective immune response may have allowed the development and maturation of the nematode in the individual’s intestine.

Humans and canines have shared the same evolutionary factors for many centuries, so the domestication of dogs led to the sharing of microbiota, pathogens and even similar eating habits^47^. This may have consequences on parasite transmission and reservoirs, such as *Strongyloides*, *Toxocara*, tapeworms and hookworms that can be passed mutually from dogs and humans^48^. Considering that population genetic studies denote a very high phylogenetic proximity between *A. caninum*, *N. americanus* and *A. duodenale*^49^, it seems reasonable that evolutionarily *A. caninum* has acquired the possibility of becoming sexually mature adult in human intestines. In fact, *A. ceylanicum* is originally a canine and feline parasite, but which over the years has acquired the ability to infect humans^11^.

Our results show the possibility of infection and reproduction of *A. caninum* in the human intestine. We suggest that additional analyses should be performed to determine the infection pathways. According to Landmann and Prociv^10^, patients with eosinophilic enteritis caused by *A. caninum* acquired the infection percutaneously. In fact, migration of the hookworm larvae into skeletal muscles has also been reported in an individual with a large cutaneous exposure, with recovery of third-stage larvae within a muscle fiber^50^. Normally, infection with this nematode occurs by the ingestion of filarioid larvae or penetration of these larvae into the skin of animals^3,4^. To complete their development, larvae need to be able to produce various enzymes involved in connective tissue degradation, such as hyaluronidases, which are often species specific^5,51,52^. Could this indicate the adaptation of the larvae of this species, which in recent times have been able to infect humans? Considering the proximity of the classical hosts to the human species, has *A. caninum* acquired the ability to develop and reproduce in the intestines of humans, or has this just not been reported so far? These are extremely important questions that may lead to new chapters in the history of parasitology.

## Methods

### Ethical statements

This work was approved by the Comitê de Ética em Pesquisa – COEP (CAAE 61047216.7.0000.5149) at the Universidade Federal de Minas Gerais (UFMG). As we used human feces obtained from commercial laboratories performing pathological analysis, an informed consent document was not required. We did not obtain any subject identification, and the data were analyzed anonymously.

### Sampling and DNA extraction

The stool samples were processed, and DNA extractions were performed for single hookworm eggs from patients collected in six Brazilian states exactly as described previously by Zuccherato and coworkers^53^. The initial isolation of the eggs was performed according to Ritchie^54^ with modifications, and samples that were positive for hookworm were stored in 10% formaldehyde for later molecular analysis. In summary, 2 ml of stool suspension was homogenized, filtered through gauze and transferred to a 15 ml tube. Five milliliters of sulfuric ether were added to the suspension and then stirred vigorously, followed by 1 min of centrifugation at 14,000 × g. The supernatant was discarded. Eggs were washed in an additional step by adding 500 μl of 1% hypochlorite for 10 min to the samples. The material was centrifuged at 14,000 × g, and the supernatant was discarded. The eggs were washed again using 500 μl of ultrapure water, followed by centrifugation at 14,000 × g. The supernatant was then discarded. The pellet was resuspended in 100 μl of ultrapure water. For DNA extraction, the eggs were observed under an optical microscope, individually pipetted into a volume of 1 μl and transferred to a 500 μl microcentrifuge tube containing 10 μl of buffer, as described by Lake and coworkers^55^. The eggs were incubated for 2 h at 57 °C, followed by incubation for 20 min at 80 °C for proteinase K inactivation. The material was stored at −20 °C until use. In total, 634 hookworm eggs from 53 patients collected in six Brazilian states were analyzed. Table 1 shows the collection sites, the number of patients and the number of eggs collected from each state.

**Table 1:** Collection sites and the numbers of patients and eggs used for the molecular identification of hookworm species.

	Number of patients	Total eggs	Mean number of eggs	Range
Bahia	6	95	15.8	14
Ceará	9	93	10.3	7
Maranhão	10	135	13.5	14
Minas Gerais	12	123	9.5	10
Piauí	10	119	11.9	12
Tocantins	6	69	7.7	14
Total	53	634	12.0	21

### Differentiation of eggs from *N. americanus* and *Ancylostoma* spp

The egg analyses for species determination were performed according to Sahimin and coworkers^16^, with some modifications. After DNA extraction from single eggs, conventional PCR was performed with three primers in the same reaction (forward NA and AD1 and reverse NC2). Table 2 shows the sequences of each primer used in this study and the respective reference. These primers were used to amplify a region in the internally transcribed spacer 2 and 28 S ribosomal RNA (ITS2-28S rRNA). The primers NA and AD1 will only anneal to DNA from *N. americanus* and *Ancylostoma* spp., respectively, while primer NC2 will anneal to DNA from many hookworm species; the NA + NC2 combination would result in a 250 bp fragment for *N. americanus* and no amplification for *Ancylostoma* spp., while the AD1 + NC2 combination would result in a 130 bp fragment for *Ancylostoma* spp. and no amplification for *N. americanus*.

**Table 2:** Primers used for the molecular determination of hookworm and host species.

Primer	Sequence (5′–3′)	Reference
AD1	CGA CTT TAG AAC GTT TCG GC	de Gruijter and coworkers^57^
NA	ATG TGC ACG TTA TTC ACT	Verweij and coworkers^58^
NC2	TTA GTT TCT TTT CCT CCG CT	Gasser and coworkers^59^
Camt1F	TGT GGC TCA AAC CAT AGC TTC	Rodrigues and coworkers^60^
Camt1R	TGT GGC ATG TCA TTA AGG GGA G	
Dmt1F	CAC ACC CAC TAC CAT CCA C	
Dmt1R	GAG GCG GTG CAT AAT GGT T	
Hmt2F	AAT CAT ACA AAG CCC CCG CA	
Hmt2R	TGG GGT TAG CGA TGG AGG TA	

All PCR amplifications were performed in a 10 μl reaction containing the three primers (NA, AD1 and NC2) (0.2 μM each), Taq DNA polymerase (1 U) (Phoneutria, Brazil), deoxynucleoside triphosphate (dNTPs) (200 μM each), reaction buffer (1X), 5 μl of single egg DNA (not previously quantified to prevent material loss) and ultrapure water. The PCR amplifications were performed according to the following program: 94 °C for 5 min, followed by 35 cycles of 94 °C for 1 min, 55 °C for 1 min and 72 °C for 1 min, and a final extension at 72 °C for 7 min. A “blank” sample was included in all amplification runs in which the DNA was replaced with water to assess the presence of possible contaminants. The reaction product was subjected to electrophoresis in a 1% agarose gel (w/v) (Midsci, USA) in 0.5X TAE buffer, and the gel was stained with GelRed™ (Biotium, USA).

### Controls

DNA from *N. americanus* and *Ancylostoma* spp. were previously extracted from adult worms^55,56^. PCRs were performed under the same conditions as previously described above with 40 ng of DNA from each parasite and the primer combinations NA + NC2 (for *N. americanus*) and AD1 + NC2 (for *Ancylostoma* spp.) (ITS2-28S rRNA). The products were then purified (Illustra GFX PCR DNA and Gel Band Purification Kit, GE Healthcare, UK), and the DNA concentration was determined. The controls fragments were subsequently cloned using the pGEM-T Easy Vector System (Promega, USA), transformed into XL1-blue cells (Phoneutria, Brazil) and recovered via minipreps (Wizard Plus Miniprep DNA Purification System, Promega, USA). The plasmids were sequenced, and the species were successfully identified. A total of 20 ng of plasmid was used as a control for the reactions for the *N. americanus* and *Ancylostoma* spp.

### Species differentiation of *Ancylostoma* spp

The molecular technique adopted in this study allowed the differentiation between the species *N. americanus* and the genus *Ancylostoma*, but for the differentiation between possible *Ancylostoma* spp. species, sequencing reactions were performed using the BigDye Terminator v3.1 Cycle Sequencing Kit (Applied Biosystems, USA) with a ABI 3130 × 1/Genetic Analyzer automated sequencer (Applied Biosystems, USA). These samples were not cloned, but were sequenced in both, forward and reverse directions, and chromatogram analysis was performed using FinchTV software (Geospiza, USA).

### Determination of possible contaminants

The fecal sample that contained *A. caninum* eggs underwent a process of DNA extraction to determine the presence of possible contamination with feces from these animals. For this, DNA extraction was performed from 220 mg of stool with the QIAamp® DNA Stool kit (Qiagen, Germany) according to the manufacturer’s recommendations. The DNA was quantified, and a total of 40 ng was used in three PCRs with different primer pairs. The Camt1F + Camt1R (167 bp), Dmt1F + Dmt1R (193 bp) and Hmt2F + Hmt2R (162 bp) primer combinations were designed to anneal only to DNA from the *Felis catus*, *Canis familiaris* and *Homo sapiens* species, respectively. These primers were used to amplify a region of the Cytochrome c oxidase subunit I (COI). Table 2 shows the sequences of each primer used and the respective reference. PCRs were performed under the same conditions as previously described above, and the sequencing of the amplified material was performed. Previously extracted DNA from *F. catus*, *C. familiaris* and *H. sapiens* were used as reaction controls.

## Supplementary information

Fig. S1.

## References

[1] Murray CJ (2012). Disability-adjusted life years (DALYs) for 291 diseases and injuries in 21 regions, 1990–2010: a systematic analysis for the Global Burden of Disease Study 2010. Lancet..

[2] Hoogerwerf MA (2019). New insights into the kinetics and variability of egg excretion in controlled human hookworm infections. J. Infect. Dis..

[3] Koide K (1961). Epidemiological observations on *Ancylostoma duodenale*, *Necator americanus* and *Trichostrongylus orientalis* infestations in Niigata Prefecture, Japan. Niigata Igakkai. Zasshi..

[4] Hasslinger MA (1986). Helminths of carnivores relevant to veterinary practice. Tierarztl. Prax..

[5] Loukas A (2016). Hookworm infection. Nat. Rev. Dis. Primers..

[6] Pan SC (2019). Cognitive and microbiome impacts of experimental *Ancylostoma ceylanicum* hookworm infections in hamsters. Sci. Rep..

[7] Prociv P, Croese J (1996). Human enteric infection with *Ancylostoma caninum*: hookworms reappraised in the light of a “new” zoonosis. Acta Trop..

[8] Croese J, Loukas A, Opdebeeck J, Fairley S, Prociv P (1994). Human enteric infection with canine hookworms. Ann. Intern. Med..

[9] Walker, N. I. *et al*. Eosinophilic enteritis in northeastern Australia. Pathology, association with *Ancylostoma caninum*, and implications. *Am. J. Surg. Pathol*. **19**(3), 328–337. 10.1097/00000478-199503000-00011 7872431

[10] Landmann JK, Prociv P (2003). Experimental human infection with the dog hookworm, *Ancylostoma caninum*. Med. J. Aust..

[11] George S (2016). Molecular identification of hookworm isolates in humans, dogs and soil in a tribal area in Tamil Nadu, India. PLoS Negl. Trop. Dis..

[12] Silva LM, Miranda RR, Santos HA, Rabelo EM (2006). Differential diagnosis of dog hookworms based on PCR-RFLP from the ITS region of their rDNA. Vet. Parasitol..

[13] Oliveira-Arbex AP (2017). Molecular identification of *Ancylostoma* species from dogs and an assessment of zoonotic risk in low-income households, São Paulo State, Brazil. J. Helminthol..

[14] Liu Y (2013). Molecular identification of *Ancylostoma caninum* isolated from cats in southern China based on complete ITS sequence. Biomed. Res. Int..

[15] Chidambaram M (2017). Evaluation of the utility of conventional polymerase chain reaction for detection and species differentiation in human hookworm infections. Trop. Parasitol..

[16] Sahimin N (2017). Hookworm infections among migrant workers in Malaysia: Molecular identification of *Necator americanus* and *Ancylostoma duodenale*. Acta Trop..

[17] Furtado LF, Rabelo ÉM (2015). Development of a new amplification-refractory mutation system for detection of a single nucleotide polymorphism linked to drug resistance in *Ancylostoma caninum*. Genet. Mol. Res..

[18] Rabelo ÉML (2017). Development of new microsatellites for the hookworm *Ancylostoma caninum* and analysis of genetic diversity in Brazilian populations. Infect. Genet. Evol..

[19] Miranda RR, Tennessen JA, Blouin MS, Rabelo EM (2008). Mitochondrial DNA variation of the dog hookworm *Ancylostoma caninum* in Brazilian populations. Vet. Parasitol..

[20] Mulinge E (2019). Molecular identification of zoonotic hookworms in dogs from four counties of Kenya. J. Helminthol..

[21] Ngui R, Lim YA, Chua KH (2012). Rapid detection and identification of human hookworm infections through high resolution melting (HRM) analysis. PLoS One..

[22] Hii SF (2018). Development and evaluation of a multiplex quantitative real-time polymerase chain reaction for hookworm species in human stool. Am. J. Trop. Med. Hyg..

[23] Gasser RB, Monti JR (1997). Identification of parasitic nematodes by PCR-SSCP of ITS-2 rDNA. Mol. Cell Probes..

[24] Ngui R, Ching LS, Kai TT, Roslan MA, Lim YA (2012). Molecular identification of human hookworm infections in economically disadvantaged communities in Peninsular Malaysia. Am. J. Trop. Med. Hyg..

[25] Monteiro KJL (2019). Mitochondrial DNA reveals species composition and phylogenetic relationships of hookworms in northeastern Brazil. Infect. Genet. Evol..

[26] Brooker S (2007). Age-related changes in hookworm infection, anaemia and iron deficiency in an area of high *Necator americanus* hookworm transmission in south-eastern Brazil. Trans. R. Soc. Trop. Med. Hyg..

[27] Marzochi MCA, Chieffi PP (1978). Studies of the factors involved in the dissemination of enteroparasites. IV. Distribution of *Necator americanus* and of *Ancylostoma duodenale* in the periurban and rural population from the municipality of Londrina, Paraná, Brazil. Rev. Inst. Med. Trop. Sao Paulo..

[28] Calvopiña M, Flores J, Guaman I, Lara G, Abarca J (2017). Chronic and severe anemia caused by *Ancylostoma duodenale* in Ecuador. Diagnosis by duodenoscopy. Rev. Chilena Infectol..

[29] Udonsi JK, Atata G (1987). *Necator americanus*: temperature, pH, light, and larval development, longevity, and desiccation tolerance. Exp. Parasitol..

[30] Tun S (2015). Detection of helminth eggs and identification of hookworm species in stray cats, dogs and soil from Klang Valley, Malaysia. PLoS One..

[31] da Silva VJ (2019). Hookworm infection aggravates metabolic disorder in obesity. Mol. Biochem. Parasitol..

[32] Furtado LFV (2019). Albendazole resistance induced in *Ancylostoma ceylanicum* is not due to single-nucleotide polymorphisms (SNPs) at codons 167, 198, or 200 of the beta-tubulin gene, indicating another resistance mechanism. Parasitol. Res..

[33] Gordon CA, Kurscheid J, Jones MK, Gray DJ, McManus DP (2017). Soil-transmitted helminths in tropical Australia and Asia. Trop. Med. Infect. Dis..

[34] Coelho WM, Amarante AF, Apolinário JC, Coelho NM, Bresciani KD (2011). Occurrence of *Ancylostoma* in dogs, cats and public places from Andradina city, São Paulo state, Brazil. Rev. Inst. Med. Trop. Sao Paulo..

[35] Reichert F (2018). Epidemiology and morbidity of hookworm-related cutaneous larva migrans (HrCLM): Results of a cohort study over a period of six months in a resource-poor community in Manaus, Brazil. PLoS Negl. Trop. Dis..

[36] Diemert DJ (2017). Safety and immunogenicity of the Na-GST-1 hookworm vaccine in Brazilian and American adults. PLoS Negl. Trop. Dis..

[37] Zhan B (2005). Biochemical characterization and vaccine potential of a heme-binding glutathione transferase from the adult hookworm *Ancylostoma caninum*. Infect. Immun..

[38] Furtado LF, Bello AC, dos Santos HA, Carvalho MR, Rabelo ÉM (2014). First identification of the F200Y SNP in the β-tubulin gene linked to benzimidazole resistance in *Ancylostoma caninum*. Vet. Parasitol..

[39] Nijsse R, Mughini-Gras L, Wagenaar JA, Ploeger HW (2014). Coprophagy in dogs interferes in the diagnosis of parasitic infections by faecal examination. Vet. Parasitol..

[40] Fahrion AS, Schnyder M, Wichert B, Deplazes P (2011). *Toxocara* eggs shed by dogs and cats and their molecular and morphometric species-specific identification: is the finding of *T. cati* eggs shed by dogs of epidemiological relevance?. Vet. Parasitol..

[41] Bradbury RS, Speare R (2015). Passage of *Meloidogyne* eggs in human stool: forgotten, but not gone. J. Clin. Microbiol..

[42] Rep BH (1972). Unfertilized hookworm eggs. Trop. Geogr. Med..

[43] Punsawad C, Phasuk N, Thongtup K, Nagavirochana S, Viriyavejakul O (2019). Prevalence of parasitic contamination of raw vegetables in Nakhon Si Thammarat Province, Southern Thailand. BMC Public Health..

[44] Duedu KO (2014). A comparative survey of the prevalence of human parasites found in fresh vegetables sold in supermarkets and open-aired markets in Accra, Ghana. BMC Res. Notes..

[45] Fuhrimann S (2015). Microbial and chemical contamination of water, sediment and soil in the Nakivubo Wetland Area in Kampala, Uganda. Environ. Monit. Assess..

[46] Liccioli S (2012). Gastrointestinal parasites of coyotes (*Canis latrans*) in the metropolitan area of Calgary. Can. J. Zool..

[47] Wang G (2013). The genomics of selection in dogs and the parallel evolution between dogs and humans. Nat. Commun..

[48] Shepherd C, Wangchuk P, Loukas A (2018). Of dogs and hookworms: man’s best friend and his parasites as a model for translational biomedical research. Parasit. Vectors..

[49] Hu M, Chilton NB, Gasser RB (2002). The mitochondrial genomes of the human hookworms, *Ancylostoma duodenale* and *Necator americanus* (Nematoda: Secernentea). Int. J. Parasitol..

[50] Little MD, Halsey NA, Cline BL, Katz SP (1983). *Ancylostoma* larva in a muscle fiber of man following cutaneous larva migrans. Am. J. Trop. Med. Hyg..

[51] Bosse M, Stoye M (1981). Effect of various benzimidazole carbamates on somatic larvae of *Ancylostoma caninum* Ercolani 1859 (Ancylostomidae) and *Toxacara canis* Werner 1782 (Anisakidae). 2. Studies of pregnant bitches. Zentralbl. Veterinarmed. B..

[52] Stone WM, Girardeau M (1968). Transmammary passage of *Ancylostoma caninum* larvae in dogs. J. Parasitol..

[53] Zuccherato LW, Furtado LF, Medeiros CS, Pinheiro CS, Rabelo ÉM (2018). PCR-RFLP screening of polymorphisms associated with benzimidazole resistance in *Necator americanus* and *Ascaris lumbricoides* from different geographical regions in Brazil. PLoS Negl. Trop. Dis..

[54] Ritchie LS (1948). An ether sedimentation technique for routine stool examinations. Bull USArmy. Med. Dep..

[55] Lake SL, Matthews JB, Kaplan RM, Hodgkinson JE (2009). Determination of genomic DNA sequences for beta-tubulin isotype 1 from multiple species of cyathostomin and detection of resistance alleles in third-stage larvae from horses with naturally acquired infections. Parasit. Vectors..

[56] Furtado LFV (2016). Standardization and application of the tetraprimer ARMS-PCR technique for screening of the E198A SNP in the β-tubulin gene of hookworm populations in Brazil. Vet. Parasitol..

[57] de Gruijter JM (2005). Polymerase chain reaction-based differential diagnosis of *Ancylostoma duodenale* and *Necator americanus* infections in human in northern Ghana. Trop. Med. Int. Health.

[58] Verweij JJ (2001). Determining the prevalence of *Oesophagostomum bifurcum* and *Necator americanus* infections using specific PCR amplification of DNA from faecal samples. Trop. Med. Int. Health.

[59] Gasser RB, Chilton NB, Hoste H, Beveridge I (1993). Rapid sequencing of rDNA from single worms and eggs of parasitic helminthes. Nucleic Acids Res..

[60] Rodrigues ACM (2017). A new whole mitochondrial genome qPCR (WMG-qPCR) with SYBR Green® to identify phlebotomine sand fly blood meals. Vet. Parasitol..

