# Supplementary material for: Egg genotyping reveals the possibility of patent Ancylostoma caninum infection in human intestine
Source: Sci Rep. 2020 Feb 20;10:3006. doi: 10.1038/s41598-020-59874-8 (PMC7033205; doi:10.1038/s41598-020-59874-8)
Supplement: Supplementary file 1 — Fig. S1. [file 41598_2020_59874_MOESM1_ESM.pdf]

**Egg genotyping reveals the possibility of patent *Ancylostoma caninum* infection in human intestine**

Luis Fernando Viana Furtado, Lucas Teixeira de Oliveira Dias, Thais de Oliveira Rodrigues, Vivian Jordania da Silva, Valéria Nayara Gomes Mendes de Oliveira, Élida Mara Leite Rabelo

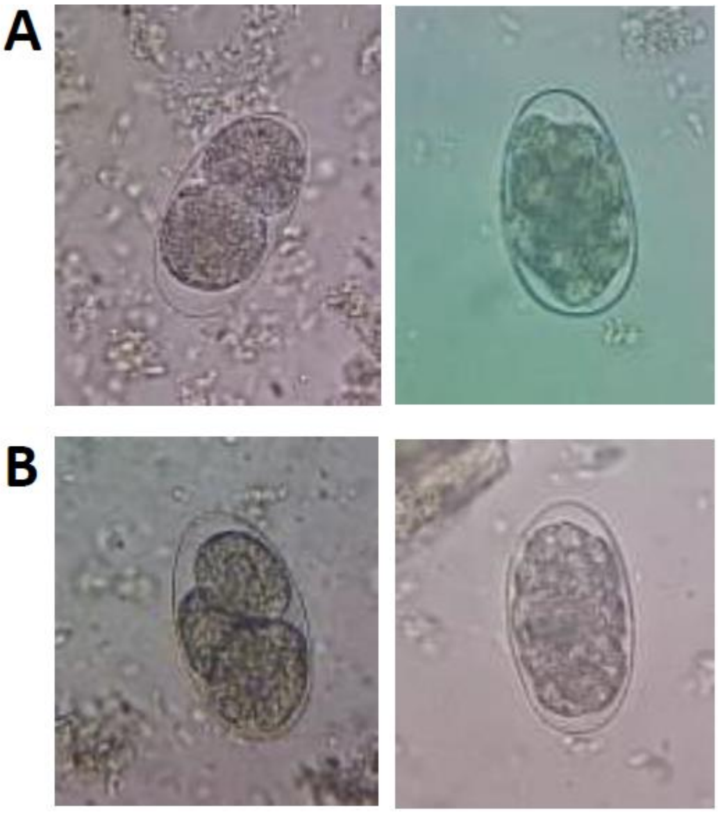


**Fig. S1.** Representative eggs of (A) *Ancylostoma caninum* eggs and (B) *Necator americanus* eggs photographed at 40X objective magnification.
